# Medical Genetics in Paraguay

**DOI:** 10.1002/mgg3.119

**Published:** 2014-11-03

**Authors:** Carlos Raúl Ferreira, María Beatriz de Herreros

**Affiliations:** 1 National Human Genome Research Institute, National Institutes of HealthBethesda, Maryland; 2 National Secretariat for the Rights of People with Disabilities (SENADIS)Fernando de la Mora, Paraguay

## Paraguay: General Statistics

**Figure 1:**
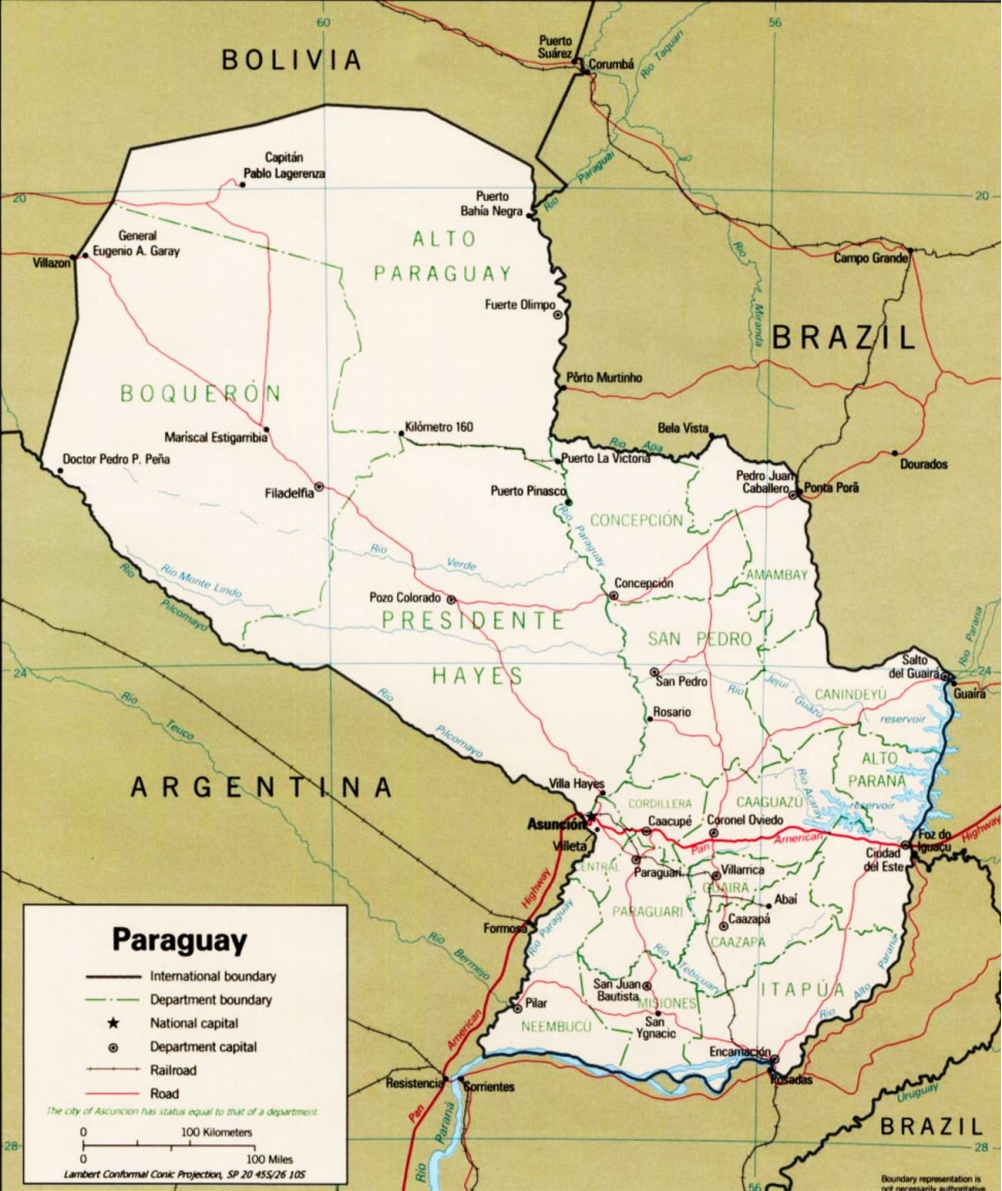
Political map of Paraguay

Paraguay is a country located in the center of South America, sharing borders with Brazil to the east and northeast, Argentina to the west and southwest, and Bolivia to the northwest. It is divided in two regions, Occidental (western) and Oriental (eastern), separated by the river Paraguay. The regions are in turn subdivided in 17 departments plus the capital district (Fig.1).

The country has a total surface area of 406,752 km^2^, roughly the same size as the state of California. The population in 2013 was 6,709,730 (General Bureau of Statistics, Surveys and Censuses, Permanent Survey of Homes 2013). The western region accounts for 60.7% of the whole territory, but according to data from the 2002 national census, only 2.6% of the population lived there. Thus, there is an uneven population distribution through the country, and the majority of people live around the capital city of Asunción. The population is fairly young, with a median age of 23.59 years (WHO Global Health Observatory, Summary of statistics).

The life expectancy at birth is 72 years for males and 78 years for females (WHO Global Health Observatory, Life tables). The GDP in 2013 was $2995 billion (World Bank, Paraguay), for a GDP per capita of $4403 (World Bank, GDP per capita). The human development index, a composite statistic of life expectancy, income and education indices, was 0.676 in 2013 – in the medium human development category – positioning the country at 111 out of 187 countries (United Nations Development Programme, Human Development Report 2014).

## Population Diversity

The Spaniards conquered the territory that would later be called Paraguay in the 1500s. Each Spanish conquistador took many native Guaraní wives, and their children were mestizos, of mixed Spanish-Guaraní race. By the end of the 16th century, the Spanish-Guaraní weddings became less common, while weddings between mestizos and criollos (born in Paraguay but of pure Spanish ancestry) were predominant. By the 1700s, the mestizos had already outnumbered the native Guaraníes. Nowadays, it is estimated that 95% of the Paraguayan population is of mestizo origin (Benitez et al. 1995).

Studies have been performed to assess if the present-day mestizos of Paraguay are genetically closer to the Spaniards or to the native Guaraníes. In one such study, HLA-A, -B, and –C polymorphisms of 50 Paraguayans (33 males and 17 females) were compared to those of Spanish and Tupí-Guaraní populations. No significant difference was found between the Paraguayan mestizos and the Spaniards, while the frequency of many polymorphisms was significantly different between the mestizos and the Tupí-Guaraníes, namely in HLA -A2, A9(23-24), A19(30-31), B5(51-52), B16(38-39), and B35 (Benitez et al. 1995). A subsequent study compared the polymorphisms in HLA-DRB1 by PCR-SSO typing of 50 Paraguayans to that of two Spanish populations and the Guaraníes. Six alleles showed significantly different frequencies between the Paraguayans the Guaraníes (DRB1*01, 06*(1314), *15, *16, and *07), while only one allele had a significantly different frequency between the Paraguayans and Spaniards (DRB1*14). This allele had a higher frequency in the Paraguayans (6%) mainly due to the presence of HLA-DRB1*1402, *1406, and *1413, all of which are rare in the Spanish population while having a higher frequency in Brazilian Guaraníes and in other South-American native tribes inhabiting near the border with Paraguay (Benitez et al. 2002). The genetic distances as calculated by the method proposed by Edwards (1971) were closer between the Paraguayan and Spanish populations (0.494 et 0.415) than between the Paraguayans and Guaraníes (0.958), suggesting a Spanish genetic predominance in modern-day Paraguayan mestizos (Benitez et al. 2002). These results were reproduced in more recent experiments conducted by the same group (Benitez et al. 2011).

There were 112,848 indigenous people living in the country in 2012 (III Census of Indigenous People, 2012), representing 1.7% of the total population. Of those, 19.0% are Mbya, 15.7% are Ava Guaraní, 14.5% are Nivaclé, and 13.4% are Paĩ Tavyterã. The rest of the indigenous people represent less than 10% each of the total indigenous population.

There is also a fairly large number of Mennonites living in Paraguay. Starting in the early 1920s, Mennonites from Canada and Eastern Europe started emigrating to the western region of Paraguay, protected by Law No 514 of 1921, or the “Mennonite law,” by which “the rights and privileges of the members of the Mennonite communities arriving to the country” were established. Nowadays, it is estimated that there are up to 40,000 Mennonites living in the country. Although no genetic disorder has been identified in the Paraguayan Mennonites so far, it is entirely likely that there are inherited disorders with a higher incidence in Paraguayan Mennonites than in the rest of the Paraguayan population. This is because a founder effect is common in Mennonite populations, as a result of the various bottleneck effects that were experienced over the course of successive waves of migration over their history. Thus, there is a high coefficient of inbreeding given the relatively small number of founder couples (Payne et al. 2011). Indeed, the Amish, Mennonite, and Hutterite Genetic Disorder Database contains 79 single-gene Mendelian disorders, with their corresponding specific mutations, that are seen in Mennonites (Payne et al. 2011). Despite the fact that the multiple advantages of studying genetic disease in the Anabaptist communities have been previously enumerated (Francomano et al. 2003), there is likely a considerable number of Paraguayan Mennonites with underlying genetic disorders that remain underserved due to a lack of diagnosis.

The prevalence of consanguinity in the country is not known (www.consang.net; accessed 09/21/2014); however, it is frowned upon in the nonindigenous population.

## Healthcare in Paraguay

In 2012, Paraguay spent 2.6 billion US$ on health care, for a total health expenditure of $392 per capita (WHO Global Health Expenditure Database, Health system financing country profile). This represents 10.32% of the GDP (WHO Global Health Expenditure Database, World Map of Total expenditure on health as % of Gross domestic product). Of the total expenditure on health in 2012, the government spent 42%, while 58% came from private sources. Social security funds represented 35% of the general government expenditure on health (WHO Global Health Expenditure Database).

Health care in Paraguay is segmented. It can be divided in the public services offered by the Ministry of Public Health and Social Welfare (MOPH), social security offered by the Institute of Social Security (IPS), and the private sector (Dullak et al. 2011). In 2013, 20.1% of the population was covered through social security, while 9.0% was covered by other types as for example private insurance, employer-based insurance, or military insurance (General Bureau of Statistics, Surveys and Censuses, Permanent Survey of Homes 2013). Even taking into account the public services offered by the MOPH, about 40% of the population lacks access to health services (Dullak et al. 2011).

In 2002, there were 6355 physicians in the country (WHO Global Health Observatory, Health workforce absolute numbers), for a density of 1.107 physicians per 1000 population (WHO Global Health Observatory, Health workforce density per 1000). There are 2.4 hospitals per 10,000 population, and 13 hospital beds per 10,000 population (WHO World Health Statistics 2014, Global Health Indicators).

There were about 160,000 births in the country in 2012; the neonatal mortality rate that year was 12 per 1000, the infant mortality rate 19 per 1000 and the under 5 mortality rate 42 per 1000 (UNICEF, Paraguay statistics). Of note, these numbers might underestimate the true mortality rates, as there is incomplete coverage of death registrations, with only 63% of deaths registered in the country according to the Training Programme on Civil Registration and Vital Statistics Systems held in Chile on November 2000, or up to 82% of deaths registered according to WHO data from 2008 (UN, Coverage of death registration).

## Genetic Testing

Medical Genetics was introduced in Paraguay by Prof. Dr. Ricardo Moreno Azorero who studied in Buenos Aires from 1961 until 1966 when he returned to Paraguay and started teaching Genetics across different colleges of the National University of Asunción (UNA). In 1974 he began working at the Health Sciences' Research Institute (IICS) where he organized a cytogenetic laboratory (Ascurra 1997). The Department of Genetics within the IICS opened its doors in 1983.

To this day, the IICS harbors the only cytogenetic laboratory in the country. It offers standard karyotype and high-resolution karyotype. The state only covers about 5% of the cost of services offered by the IICS, despite the fact that it is a public institution. Thus, the coverage is very low as only a small percentage of the population is able to afford the costs of these services (Ascurra 2004).

Chromosomal microarrays are not performed in the country, although samples can be sent out of the country, assuming that patients can afford the cost of the test, and the cost of shipping. There is no Preimplantation Genetic Diagnosis center.

Recently, a laboratory offering clinical molecular tests became available (Genext), although reportedly the tests are again sent out of the country.

There is also no laboratory specialized in metabolic tests, and these need to be sent to countries such as Chile, Brazil or Argentina.

## Genetic Services in Paraguay

Law No 780 from 1979 created the National Institute for the Protection of People with Special Needs (INPRO), that in 2012 changed its name to National Secretariat for the Rights of People with Disabilities (SENADIS). Although the SENADIS is not just dedicated to patients with genetic disorders but to people with disabilities in general, the only accredited clinical geneticist in the country – one of the coauthors of this manuscript, M.B.H. – provides regular genetic care at this institute.

A total of 2288 were seen at the Medical Genetics clinic at the INPRO (now SENADIS) between December 2004 and December 2009, an average of 38 patients per month. The distribution by gender was 1326 males (58%) while 962 were females (42%). The distribution according to age was 1265 patients between 0 and 4 years old (55.3%), 567 between 5 and 9 years old (24.8%), 270 between 10 and 14 years old (11.8%), 120 between 15 to 19 years old, and 66 (5.2%) patients 20 years old and older (2.9%).

Aside from the aforementioned Department of Genetics at the IICS and the Medical Genetics clinic at the SENADIS, there is no hospital with a dedicated Genetics department, although the one clinical geneticist acts as a consulting service when required.

In addition to the aforementioned clinical geneticist, there is also a gynecologist working at the IPS whose focus is on prenatal and reproductive genetics.

## Genetics Training

Medical Genetics is not formally part of the curriculum during medical school, and there are at the most a few hours of Genetics classes over the course of a 6-year medical education.

There is also no postdoctoral Medical Genetics training in the form of a residency or fellowship program. Physicians who want to pursue a career in Genetics need to train in other countries.

There are no genetic counselors in the country, and training in genetic counseling is not provided in the country. Thus, genetic counseling through the country is provided either by a geneticist, pediatricians, family physicians, or gynecologists.

## Prevalence of Mendelian and Chromosomal Disorders

The estimated birth prevalence for recessive single-gene disorders is 1.7 per 1000 live births, given 289 patients born with recessive disorders out of 170,000 total births in 2001 (March of Dimes Global Report on Birth Defects, 2006). Using a model that predicts the prevalence of dominant single-gene disorders at 7 per 1000 live births, and that of X-linked single-gene disorders at 1.3 per 1000 live births, the estimated number of births of children affected with dominant single-gene disorders is 1190 per year, while for X-linked single-gene disorders it is 221 affected births per year (March of Dimes Global Report on Birth Defects, 2006).

The birth prevalence of Down syndrome in Paraguay has been estimated at 1.98 per 1000 in the period between 1998 and 2005, according to data from the Latin American Collaborative Study of Congenital Malformations, or ECLAMC (Nazer and Cifuentes 2011a). This number is similar to the one obtained by the March of Dimes, with a birth prevalence of 1.7 per 1000 live births, given 289 patients born with Down syndrome out of 170,000 total births in 2001 (March of Dimes Global Report on Birth Defects, 2006). That same year it is estimated that there were 306 children born with sex chromosome disorders, and 85 children born with other chromosomal disorders, for a total of 680 births affected with a chromosomal disorder per year (March of Dimes Global Report on Birth Defects, 2006).

## Burden of Congenital Malformations in Paraguay

In neonates and in children under 5 years of age, congenital anomalies represent the second most common cause of death in the country, behind prematurity. In 2012, congenital anomalies accounted for 22.1% of all causes of death in newborns, and 20.7% of all causes of death in children under 5 years of age (WHO Global Health Observatory, Distribution of causes of death among children). It is estimated that congenital anomalies accounted for 800 deaths in the country in 2012 (WHO Global Health Estimates, Disease country mortality estimates 2012) and the burden of disease from congenital anomalies that same year was estimated at 75,600 disability-adjusted life years (DALYs), 4.1% of the total DALYs for the country (WHO Global Health Estimates, DALY estimates 2012).

In 2001 there were 9342 children born with a birth defect in the country, out of 170,000 total births, for a prevalence of 55.0 per 1000 live births (March of Dimes Global Report on Birth Defects, 2006). Of those, 6290 represented children born with malformations not related to chromosomal or single-gene disorders. In turn, 1479 were born with defects of the musculo-skeletal system, 1343 were born with malformations of the cardiovascular system, 1275 with malformations of the genitalia, 476 with malformations of the digestive system, 340 with neural tube defects, 221 with other birth defects of the central nervous system, 272 with defects of the urinary tract, 238 with facial clefts, 85 with malformations of the ear, face or neck, 51 with eye defects, 51 with malformations of the respiratory system, and 459 with other and multiple birth defects (March of Dimes Global Report on Birth Defects, 2006).

Data from the Latin American Collaborative Study of Congenital Malformations (ECLAMC) between 1995 and 2008 revealed a prevalence per 10,000 births of 0.4 for renal agenesis, polycystic kidney 1.9, hydronephrosis 3.7, hypospadias 3.4, ambiguous genitalia 2.6, diaphragmatic hernia 2.2, cleft lip 14.6, cleft palate 4.5, anencephaly 7.8, microcephaly 2.2, anotia/microtia 3.0, hydrocephalus 13.8, cephalocele 2.2, duodenal atresia 2.2, esophageal atresia 3.0, imperforate anus 4.1, gastroschisis 4.5, omphalocele 3.7, arthrogryposis 1.9, hip dysplasia 10.1, club foot 18.9, polydactyly 20.9, syndactyly 4.9, and congenital heart disease 29.8 per 10,000 births (Nazer and Cifuentes 2011b).

The prevalence of neural tube defects is 2.0 per 1000 live births (March of Dimes Global Report on Birth Defects, 2006). Folic acid enrichment is mandated by law. Decree 20830 from 1998 declares obligatory the enrichment of wheat flour with iron and vitamins, including folic acid (INAN, Decree 20830/98). The National Institute of Food and Nutrition (INAN) regulates the amount of folic acid at 3.0 mg per kilogram of wheat flour (INAN, Resolution 27 from 2002).

There is no official registry for congenital malformations in the country. However, since 1975 the Neonatology unit from the Hospital de Clínicas (the national public hospital) and later the Neonatology unit from the Paraguayan Red Cross became part of the Latin American Collaborative Study of Congenital Malformations, or ECLAMC (Ascurra 1997).

Agriculture, and in particular soy cultivation, is one of the main sources of revenue for the country, leading to a massive use of pesticides. More than 24 million liters of toxic agricultural chemicals are used each year across soy fields in Paraguay (Benítez-Leite et al. 2007). A prospective case–control study performed in Encarnación, Itapúa between March 2006 and February 2007 found an association between exposure to pesticides and congenital malformations. The risk factors that reached statistical significance were living close to fumigated fields (OR = 2.46, 95% CI 1.09–5.57, *P* < 0.02), living specifically less than 1 km away from the fumigated fields (OR = 2.66, 95% CI 1.19–5.97, *P* < 0.008), and storing pesticides inside the house (OR = 15.35, 95% CI 1.96–701.63, *P* < 0.003) (Benítez-Leite et al. 2007).

## Reproductive Law

A retrospective review of 300 amniotic fluid samples obtained over the course of 6 years at the IICS found a chromosomal anomaly in 4% of cases (Ascurra et al. 2002). However, the abortion policy in the country, enacted by the Paraguayan Penal Code (Law No 1160 of 1997), prohibits abortions to be performed in cases of fetal impairment (such as chromosomal anomalies or congenital malformations). It also prohibits abortions to be performed in order to preserve the physical or mental health of the woman, in cases of rape or incest, or for economic or social reasons. It only allows an abortion to be performed in order to save the life of a woman endangered by pregnancy or childbirth (UN – Abortion policy in Paraguay).

Anyone performing an abortion, including a woman who causes her own abortion or simply consents to it, is subject to 15–30 months of imprisonment. Despite the severity of the law, clandestine abortion is quite common in Paraguay, and it is estimated that there are 26,000 illegal abortions performed in the country each year, and that 35% of women have had at least one abortion during their lifetimes (UN – Abortion policy in Paraguay). This is a serious national health problem, as according to one study by the Center of Human Rights in Paraguay (Codehupy) and the Center for Documentation and Studies (CDE) between 1996 and 2009, 23% of all maternal deaths were related to abortion, with an average of 30 deaths occurring each year as a complication of a prior abortion (Abortion, penal system and women's human rights, 2013).

Despite the apparent severity of the law, it should be noted that between 2006 and 2010 there was an average of 8343 women admitted to the hospital each year as a result of abortions, 4351 consults per year secondary to abortion, and only eight women imprisoned during the 5-year period as a result of it, only two of which were imprisoned for more than 1 year (Abortion, penal system and women's human rights, 2013).

Misoprostol is still used in the country with the purpose of inducing illegal abortions, and it has been associated with Moebius sequence, transverse limb defects, and arthrogryposis (Herreros et al. 2003, 2009).

## Newborn Screening

Newborn screening activities began in the private sector in 1995, and could be obtained by parental request (Borrajo 2007). However, the pilot national newborn screening program for congenital hypothyroidism did not start until 1999.

Law No 2138 from 2003 created the national Program for the Prevention of Cystic Fibrosis and Mental Retardation (PPFQRM), with the goals of ensuring therapy and follow-up for each disorder detected, reducing morbidity and mortality, providing guidelines for each disorder, as well as collecting epidemiological data. It was incorporated as a Program of the MOPH by Decree No 2126 in 2004. Currently, the PPFQRM offers mandatory universal screening for congenital hypothyroidism and phenylketonuria (PKU), while screening for cystic fibrosis is offered selectively.

The samples are obtained as dried blood spots through a heel stick between 40 hours and 7 days of life. In cases of preterm birth at <35 weeks gestation the sample is obtained at 7 days of life and is repeated at 22 days of life. Newborns discharged at an early time, before 40 hours of life, are not tested for PKU, only for CH. (PPFQRM, Guide).

Its scope covers the hospitals and health centers belonging to the MOPH, where around 55% of the babies are born (Borrajo 2007). In cases where a birth occurred at a center lacking the infrastructure to obtain the samples, or in cases of home births, it is the responsibility of the parents or the primary caregivers to transport the baby before 7 days of life to a facility where the dried blood spot sample can be obtained (PPFQRM, Guide).

The government provides funding, but this is insufficient to cover all the costs of the program, that receives support from international agencies of cooperation (Borrajo 2007).

There are no national guidelines on newborn hearing screening.

The number of samples analyzed through the PPFQRM in the last 7 years is shown in Table1.

**Table 1:** Number of newborn screening samples per year (data from http://piecito.org/web/)

Year	Number of samples analyzed
2008	36,520
2009	44,095
2010	55,370
2011	66,162
2012	73,293
2013	73,350
2014 (until July)	56,698

Data presented at the 12th Paraguayan Congress of Pediatrics in October 2010 revealed that out of 291 pregnant women, 43% did not know about newborn screening (PPFQRM, Survey about Newborn Screening Knowledge). A subsequent survey conducted after more widespread media coverage revealed that knowledge about the test had increased to 80% (PPFQRM, Evaluation of Newborn Screening coverage in Paraguay). As can be seen from the data shown above, both the coverage and knowledge of newborn screening continues to increase in the country.

The analyte used for the detection of congenital hypothyroidism is TSH, measured by the AutoDELFIA automatic immunoassay system (Perkin Elmer Life Science, Turku, Finland), with a cutoff of 10 *μ*U/mL. This cutoff was validated after analyzing 20,168 newborn dried blood spot samples, with the 99th percentile found at 9 *μ*U/mL (PPFQRM, TSH reference values in Paraguayan newborns). If the level is above the cutoff and the sample was obtained before 48 hours of life, then the sample is repeated; if the level was obtained after 48 hours of life, or if TSH is >50 *μ*U/mL regardless of the time at which the sample was obtained, then the newborn will be referred to a specialist (PPFQRM, Guide). Between October 2008 and August 2011 there were 79 patients with congenital hypothyroidism under treatment out of 154,705 newborns screened, for an incidence of 1/1958 (PPFQRM, Evaluation of Newborn Screening coverage in Paraguay).

Iodine deficiency – leading to hypothyroidism – used to be a widespread problem in the country before laws were put in place to address the problem. Article 183 from Act 836 of the Sanitary Code from 1980 establishes that salt destined to human consumption should be iodized. The National Institute of Food and Nutrition (INAN) regulates the amount of iodine in salt at 30 to 50 mg of iodine per kilogram of salt (30–50 ppm) in the form of 51 to 84 mg of potassium iodate (KIO_3_) per kilogram of salt (INAN, Resolution No 163 from 2009). The iodized salt consumption rate between 2008 and 2012 was 93.4% (UNICEF, Paraguay statistics).

Reduction or even elimination of salt consumption is part of the treatment for hypertension. Interestingly, a Paraguayan study of 68 mothers of children born with congenital hypothyroidism found that 22 of them (32.3%) had hypertension during pregnancy, with 17 of them completely eliminating salt from their diet and five reducing the amount of salt. None of these 22 pregnant women received additional iodine supplementation despite the fact that recommended dietary intake of iodine doubles during pregnancy (Ascurra et al. 2013). Although this study was not performed to detect a statistical association between gestational hypertension and congenital hypothyroidism, it seems reasonable to recommend an alternative form of iodine supplementation in Paraguayan women with hypertension during pregnancy.

The screening technology used for PKU/hyperphenylalaninemia is a fluorescent ninhydrin method, with a cutoff of >2 mg/dL. Newborns with hyperphenylalaninemia above the cutoff get periodic monitoring of phenylalanine levels, and a diagnosis of PKU is given with values >6 mg/dL. A cross-sectional study of clinical cases diagnosed with hyperphenylalaninemia detected between October 1999 and July 2013 revealed 39 cases out of a total of 379,517 newborns tested, 19 of which were confirmed to have PKU, for an incidence of 1/19,975 (Ortiz-Paranza et al. 2013).

Cystic fibrosis is screened by measuring immunoreactive trypsinogen (IRT), with a cutoff level of >70 ng/mL. If levels are above the cutoff, a second sample is obtained, and if the values are again >70 ng/mL then a sweat test is obtained. In infants more than 30 days old, a sweat test is obtained directly without measuring the IRT (PPFQRM, Guide). Between October 2008 and August 2011 there were four cases of cystic fibrosis detected out of 15,650 screened, for an incidence of 1/3912 (PPFQRM, Evaluation of Newborn Screening coverage in Paraguay).

It is not known how many inborn errors of metabolism are missed through the country, and the reasons for this are manifold. First, many physicians taking care of these patients are unlikely to recognize the symptoms associated with these disorders, as the only exposure they got to Genetics amounted to a few hours over the course of medical school. Second, even in the case that the keen physician were to suspect the diagnosis of an inborn error of metabolism, there is no laboratory in the country that would offer a clinical test for the diagnosis of such disorder. One might still send out the test out of country, but in many cases the results won't be available in a clinically relevant timeframe. Even assuming that the diagnosis is made, the lack of availability of laboratory tests also makes it difficult in many instances to monitor the course of the disease. Lastly, medical formulas for specific inborn errors of metabolism are not currently available in the country. Although no cost-utility analysis has been performed, it is likely that screening for at least some inborn errors of metabolism would be cost-effective -as it has been shown in other countries (Pandor et al. 2004; Carroll and Downs 2006; Norman et al. 2009) – even when taking into account the cost of equipment, service contract, reagents, personnel, treatment, as well as the inflation rate. However, such a cost-utility analysis would be hard to perform being that the prevalence for the majority of inborn errors of metabolism is not known in the country, for reasons that were already mentioned. Finally, even assuming that the newborn screening program is expanded to other inborn errors of metabolism, and that there is an infrastructure in place for the confirmation of diagnosis, monitoring of the progression and treatment of the disease, there would still be a need for more clinical geneticists in order to take care of these newly diagnosed patients, as there are currently no specialists in metabolic diseases in the country.

## Conclusions

Given that medical resources are mostly pooled around the capital city of Asunción, the use of telemedicine in the form of telegenetics (Hilgart et al. 2012) might represent a useful tool in the evaluation of patients with suspected genetic disorders, although it will still not represent a solution to the problem of the paucity of clinical geneticists in the country. Indeed, Paraguay is extremely underserved when it comes to clinical genetic care, with only one clinical geneticist available for a country of almost seven million people. This scenario is unlikely to improve in the short term, as Medical Genetics is not part of the core curriculum in medical school, and there is no postdoctoral training in Genetics anywhere in the country. A first step toward a long-term solution to this problem would be to fully incorporate genetic education within the medical school curriculum by adapting models used in other curriculums around the world (Robinson and Fong 2008) to our local situation, followed by the institution of an accredited postdoctoral residency training program in genetics.

## Biosketches

Carlos Ferreira, M.D. is a native of Paraguay, went to medical school at the National University of Asunción, completed an Internal Medicine residency at Rush University Medical Center in Chicago, Illinois, and a fellowship in Clinical Genetics at the National Human Genome Research Institute and Johns Hopkins Hospital Medical Genetics consortium. Currently, he's pursuing a subspecialty in medical biochemical genetics.

María Beatriz de Herreros, M.D. obtained her medical degree from the National University of Asunción in 1983. From 1995 until 2006, and again from 2009 until 2011 she was a staff member at the Department of Genetics and Molecular Biology of the IICS. Since 2004 she has been in charge of the Genetics outpatient clinic at the INPRO/SENADIS. She's a founding member of the Society for Prenatal Diagnosis of Paraguay (SODIAP), a member of the drafting committee for the journal Progress in Prenatal Diagnosis and Treatment, and the Paraguayan professional delegate for the International Prader-Willi Syndrome Organisation.

## Websites

Abortion, penal system and women's human rights, 2013: http://www.cde.org.py/web/attachments/article/154/Aborto,%20sistema%20penal%20y%20DDHH%20de%20las%20mujeres.pdf. Accessed 09/21/2014.

Amish, Mennonite and Hutterite Genetic Disorder Database: http://www.biochemgenetics.ca/plainpeople/view.php. Accessed 09/21/2014.

III Census of Indigenous People, 2012: http://www.dgeec.gov.py/Publicaciones/Biblioteca/censo%20indigena%202012/Presentacion%20resultados%2019%2007%2013.pdf. Accessed 09/21/2014.

INAN – Decree 20830/08: http://www.inan.gov.py/docs/Decreto%2020830%201998.pdf. Accessed 09/21/2014.

INAN - Resolution No 163 from 2009: http://www.inan.gov.py/docs/res163.pdf. Accessed 09/21/2014.

INAN, Resolution No 27 from 2002: http://www.aladi.org/nsfaladi/normasTecnicas.nsf/09267198f1324b64032574960062343c/8963f339b170aa11032579e600493cde/$FILE/Resolución%20No%2027-2002.pdf. Accessed 09/21/2014.

March of Dimes Global Report on Birth Defects, 2006: http://www.marchofdimes.org/mission/march-of-dimes-global-report-on-birth-defects.aspx. Accessed 09/21/2014.

United Nations Development Programme - Human Development Report 2014: http://hdr.undp.org/sites/all/themes/hdr_theme/country-notes/PRY.pdf. Accessed 09/21/2014.

General Bureau of Statistics, Surveys and Censuses - Permanent Survey of Homes 2013: http://www.dgeec.gov.py/Publicaciones/Biblioteca/EPH2013/PUBLICACION%20EPH%202013.pdf. Accessed 09/21/2014.

PPFQRM – Evaluation of Newborn Screening coverage in Paraguay: http://piecito.org/web/wp-content/uploads/Evaluación-de-la-Cobertura-de-la-Detección-Neonatal-en-el-Paraguay.pdf. Accessed 09/25/2014.

PPFQRM – Guide: http://piecito.org/web/wp-content/uploads/2010/03/guia-Toma-de-Muestra.pdf. Accessed 09/25/2014.

PPFQRM – Survey about Newborn Screening Knowledge: http://piecito.org/web/wp-content/uploads/Encuesta-Sobre-Conocimiento-del-Estudio-de-Detección-Neonatal-test-del-Piecito-en-Embarazadas-Concurrentes-a-Servicios-de-Salud-Publica.pdf. Accessed 09/25/2014.

PPFQRM, TSH reference values in Paraguayan newborns: http://piecito.org/web/wp-content/uploads/Valor-De-Referencia-De-La-Hormona-Estimulante-De-La-Tiroides-TSH-Por-Fluoroinmunoensayo-En-Neonatos-Del-Paraguay.pdf. Accessed 09/25/2014.

UN – Abortion policy in Paraguay: www.un.org/esa/population/publications/abortion/doc/paraguay.doc. Accessed 09/21/2014.

UN – Coverage of death registration: http://unstats.un.org/unsd/demographic/CRVS/Website_final_coverage.xls. Accessed 09/28/2014.

UNICEF – Paraguay statistics: http://www.unicef.org/infobycountry/paraguay_statistics.html. Accessed 09/21/2014.

WHO Global Health Estimates - DALY estimates 2012: http://www.who.int/entity/healthinfo/global_burden_disease/GHE_DALY_2012_country.xls?ua=1. Accessed 09/28/2014.

WHO Global Health Estimates - Disease country mortality estimates 2012: http://www.who.int/entity/healthinfo/global_burden_disease/GHE_Deaths_2012_country.xls?ua=1. Accessed 09/28/2014.

WHO Global Health Expenditure Database: http://apps.who.int/nha/database/ViewData/Indicators/en. Accessed 09/21/2014.

WHO Global Health Expenditure Database – Health system financing country profile: Paraguay 2012: http://apps.who.int/nha/database/Country_Profile/Index/en. Accessed 09/21/2014.

WHO Global Health Expenditure Database – World Map of Total expenditure on health as % of Gross domestic product: http://apps.who.int/nha/database/World_Map/Index/en?id=REPORT_4_WORLD_MAPS&mapType=3&ws=0. Accessed 09/21/2014.

WHO Global Health Observatory - Distribution of causes of death among children: http://apps.who.int/gho/data/view.main.ghe300-PRY?lang=en. Accessed 09/28/2014.

WHO Global Health Observatory - Health workforce absolute numbers: http://apps.who.int/gho/data/node.main.A1443?lang=en. Accessed 09/28/2014.

WHO Global Health Observatory - Health workforce density per 1000: http://apps.who.int/gho/data/node.main.A1444?lang=en. Accessed 09/28/2014.

WHO World Health Statistics 2014 – Part III. Global Health Indicators:

http://who.int/gho/publications/world_health_statistics/EN_WHS2014_Part3.pdf?ua=1. Accessed 09/21/2014.

World Bank - Paraguay: http://data.worldbank.org/country/paraguay. Accessed 09/21/2014.

World Bank – GDP per capita: http://data.worldbank.org/indicator/NY.GDP.PCAP.CD. Accessed 09/21/2014.

## Conflict of Interest

None declared.
