# Supplementary figures and images for: Medical Genetics in Paraguay
Source: Mol Genet Genomic Med. 2014 Nov 3;2(6):458–66. doi: 10.1002/mgg3.119 (PMC4303215; doi:10.1002/mgg3.119)

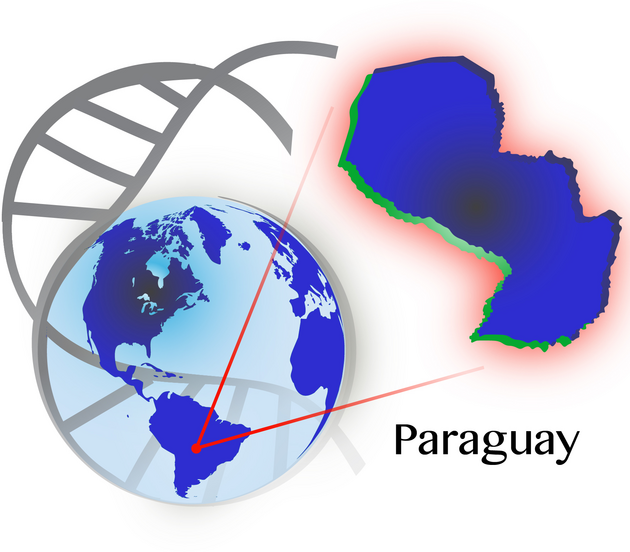

Supplement: Supplementary file 1 [file mgg30002-0458-fu1.png]
